# The worst and the best: new insights into risk and resilience in young adults from the COVID-19 pandemic

**DOI:** 10.1007/s42844-023-00096-y

**Published:** 2023-04-27

**Authors:** Lilly Shanahan, Lydia Johnson-Ferguson, Michelle Loher, Annekatrin Steinhoff, Laura Bechtiger, Aja Louise Murray, Urs Hepp, Denis Ribeaud, Manuel Eisner

**Affiliations:** 1 Jacobs Center for Productive Youth Development, University of Zurich, Andreasstrasse 15, 8050 Zurich, Switzerland; 2 Department of Psychology, University of Zurich, Binzmühlestrasse 14, 8050 Zurich, Switzerland; 3 Experimental and Clinical Pharmacopsychology, Department of Psychiatry, Psychotherapy, and Psychosomatics, Psychiatric University Hospital Zurich, University of Zurich, Lenggstrasse 31, 8032 Zurich, Switzerland; 4 University Hospital of Child and Adolescent Psychiatry and Psychotherapy, University of Bern, Bern, Switzerland; 5 Department of Psychology, University of Edinburgh, Edinburgh, UK; 6 Meilen Institute Zurich, Stockerstrasse 45, CH-8002 Zurich, Switzerland; 7 Institute of Criminology, University of Cambridge, Sidgwick Avenue, Cambridge, CB3 9DA UK

**Keywords:** COVID-19, protective factors, risk, resilience, young adulthood

## Abstract

Historic declines in young people’s mental health began to emerge before the COVID-19 pandemic. In the face of this youth mental health crisis, the pandemic constituted a naturalistic stressor paradigm that came with the potential to uncover new knowledge for the science of risk and resilience. Surprisingly, approximately 19-35% of people reported better well-being in the first few months of the COVID-19 pandemic than before. Therefore, in May and September 2020, we asked *N*=517 young adults from a cohort study to describe the best and the worst aspects of their pandemic lives (*N*=1,462 descriptions). Inductive thematic analysis revealed that the best aspects included the deceleration of life and a greater abundance of free time, which was used for hobbies, healthy activities, strengthening relationships, and for personal growth and building resilience skills. Positive aspects also included a reduction in educational pressures and work load and temporary relief from climate change concerns. The worst aspects included disruptions and changes to daily life; social distancing and restrictions of freedoms; negative emotions that arose in the pandemic situation, including uncertainty about the future; and the growing polarization of society. Science that aims to reverse the youth mental health crisis must pay increased attention to sources of young people’s distress that are not commonly measured (e.g., their educational, work, and time pressures; their fears and uncertainties about their personal, society’s, and the global future), and also to previously untapped sources of well-being – including those that young people identified for themselves while facing the COVID-19 pandemic.

**Supplementary Information:**

The online version contains supplementary material available at 10.1007/s42844-023-00096-y.

## Introduction

The field of risk and resilience research aims to identify risk factors, or “stressors,” that increase a person’s probability of developing psychopathology (Kraemer et al., 1997). It also strives to identify individual and contextual protective factors that contribute to better-than-expected well-being (i.e., resilience) in the face of such risks (e.g., Rutter, 2013). Classic studies of resilience have identified notable protective factors (e.g., Masten, 2001; Rutter, 1990; Werner, 1993). Modifiable ones include warm and supportive social relationships, adaptive self-regulation, and self-efficacy, for example. Less easily modifiable protective factors include being likeable, having an “easy” temperament, cognitive skills and intelligence, and higher socioeconomic status (Masten et al., 2021).

Progress in risk and resilience research has been notable as the field expanded from delineating the phenomenon of resilience to developing interventions based on identifiable protective factors to improve people’s well-being (Masten et al., 2021). Despite this progress, the mental health of adolescents and young adults in the Western world has declined since before the COVID-19 pandemic, with adolescents’ and young adults’ rates of depression and anxiety reaching historically high levels (e.g., Keyes et al., 2019; Twenge et al., 2019). Similarly, their rates of self-destructive behavior, such as non-suicidal self-injury, suicidal behavior, and drug overdoses, reached all-time highs (e.g., Monto et al., 2018; Patalay & Gage, 2019). As the pandemic became a chronic stressor, many young people’s mental health issues were aggravated (e.g., Racine et al., 2021). In response, leading health officials, including the U.S. Surgeon General, declared a mental health crisis among young people (Office of the U.S. Surgeon General, 2021).

The causes of the upsurge in young people’s mental health problems – even before the pandemic – are not fully understood. Declines in economic and labor market prospects likely undermine mental health, and the role of increased use of social and digital media is being clarified (e.g., Muller et al., 2020; Odgers & Jensen, 2020; Twenge et al., 2020). Arguably, the pace of insights from the field of risk and resilience has not kept up with the pace of declines in mental health. Indeed, the field invariably examines the same set of risk factors, including, for example, poverty, maltreatment, or family history of mental health problems (Franklin et al., 2017), and, similarly, the same modifiable protective factors (e.g., social support). While these factors are important, their repeated investigation may not greatly enhance insights into young people’s mental health development (Franklin et al., 2017).

How can we accelerate the pace of gaining new conceptual insights for the field of risk and resilience research? Young people themselves could help generate new knowledge (Luthar et al., 2021). For example, youth can describe the factors that enhance or dampen their well-being – in their own words – rather than on pre-defined measures. Young people’s answers can then be analyzed, using bottom-up techniques, to identify the key themes that are common to their answers (Braun & Clarke, 2006). Newly identified themes can then inform new insights into risk and protective processes in mental health development, and also serve as the basis for developing new measures for quantitative studies in risk and resilience research.

## The COVID-19 pandemic as an opportunity to advance the science of risk and resilience

The COVID-19 pandemic constituted a novel stressor faced by virtually all young people beginning in 2020. This naturalistic stressor paradigm also provided a unique opportunity to advance the science of risk and resilience by observing how young people perceived and coped with this new situation (Roubinov et al., 2020). Surprisingly, several studies conducted in the first few months of the pandemic revealed that a sizable minority of people (typically between 19 and 35%) reported better mental health during the pandemic than before (e.g., Luthar et al., 2021; Panzeri et al., 2021; Penner et al., 2021; Shanahan et al., 2022; Silk et al., 2021; Soneson et al., 2022). Indeed, in some of these studies, only a minority of participants reported significantly worse well-being during the pandemic than before. But what accounted for improved or stable mental health during the first few weeks of the pandemic despite the presence of a potentially severe stressor that undoubtedly disrupted people’s lives? Insights into this question could provide important new lessons for risk and resilience research.

We surveyed young adults in Zurich, Switzerland, twice: in late May, 2020, after the end of the first lockdown, and again in mid-September, 2020, after a relatively COVID- and restrictions-free summer. What did summer 2020 look like Switzerland in terms of COVID-19-related measures and restrictions? In June 2020, night clubs, cinemas, theatres, and public spaces (temporarily) reopened. Gatherings of groups with more than five people were permitted again, including in restaurants. Sports facilities and swimming pools reopened. Select educational institutions for older adolescents and young adults (e.g., vocational schools) reopened (Kohler et al., 2020). Beginning in late June, events for up to 300 people were allowed to take place again (although this option was not exercised by many event organizers). In sum, many COVID-19-related restrictions that had limited young adults’ social lives were (temporarily) lifted in the summer of 2020, although the Swiss government urged the population to remain cautious.

At both measurement points, we asked young adults what they experienced as the best and the worst aspects of their lives during the pandemic. Switzerland, like many places, had also seen an emerging youth mental health crisis since before the pandemic: Similar to the US, depressive symptoms among young people in Switzerland had increased for more than a decade before the pandemic (Schweizerisches Gesundheitsobservatorium [Swiss Health Observatory], 2020). Furthermore, there was a strong increase in psychiatric in- and outpatient services use in this age-group (Schuler et al., 2017). Our study analyzed young adults’ responses about their well-being during the pandemic using inductive thematic analysis (Braun & Clarke, 2006). The ultimate aim of this study was to generate new insights that can also inform quantitative research on risk and resilience going forward.

## Methods

### Sample and procedures

Data came from the Zurich Project on the Social Development from Childhood to Adulthood (z-proso), a prospective-longitudinal study (Ribeaud et al., 2022). The study recruited children who entered 1^st^ grade in one of 56 public primary schools in Zurich in 2004. The initial target sample of schools was selected using random sampling procedures (slightly oversampling disadvantaged school districts). Consistent with Switzerland’s immigration policies and Zurich’s diverse population, parents of participants were born in over 80 countries. Parental educational background was diverse: 26.2% of families had more than one parent with a university degree. The mean household International Socioeconomic Index (ISEI) of occupational status (Ganzeboom et al., 1992) score was 45.74 (SD=19.24). This is an internationally comparable index of socioeconomic status based on occupation-specific income and required educational level [range = 16 (e.g., unskilled worker) to 90 (e.g., judge)].

The original study consisted of eight assessment waves, at ages 7, 8, 9, 11, 13, 15, 17, and 20 (Ribeaud et al., 2022). In April 2020, all participants (mean age 22.5 years old) who had participated in the age 20 assessment in 2018 (*N*=1,180) were invited to participate in an online COVID-19 study. Of the eligible participants, *n*=21 could not be reached due to invalid contact information or unclear status. Out of *n=*1,159 participants contacted, *n*=786 responded to the mid-April 2020 survey within one week (67.8% of age 20 sample). Respondents in the age 22 COVID-19 surveys were more likely to be female and to come from a non-migrant background compared to those who participated in the first z-proso assessment at age 7 (see also, Shanahan et al., 2022). In total, four COVID-19 data collections took place in mid-April (*n*=786), early May (*n*=650), late May (*n*=569), and mid-September 2020 (*n*=525). We used the last two COVID-19 surveys for the current analysis.

Open-ended questions about young adults’ best and worst experiences during the COVID-19 pandemic were collected twice, during the late May and the mid-September 2020 data collections. These questions were added to our COVID-19 assessment batteries in response to results from our initial lockdown data collection, which revealed that almost one in five participants reported better well-being during the first few weeks of the lockdown than before the pandemic (Shanahan et al., 2022). To investigate this finding, we added the open-ended question to the May 21-27 2020 assessment, which took place after the first COVID-19 lockdown, and to the September 10-16 2020 assessment, which took place after a relatively measures- and restrictions-free summer.

A total of *n*=517 participants completed at least one of the open-ended questions during these assessments. The total number of qualitative statements was *N*=1,462. The online COVID-19 surveys required approximately 15 to 20 minutes to complete; participants were entered into a lottery to win one of 50 prizes of about $100. Participants provided written online informed consent for their study participation. Ethical approval was obtained by the Ethics Committee of the Faculty of Arts and Social Sciences at the University of Zurich. The authors assert that all procedures contributing to this work comply with the ethical standards of the relevant national and institutional committees on human experimentation and with the Helsinki Declaration of 1975, as revised in 2008. For a timeline of the COVID-19 pandemic in Switzerland during our study period, see p. 564 of Steinhoff et al. (2021).

### Measures

Participants were asked two open-ended questions at the end of the late-May and mid-September COVID-19 surveys, respectively. In May, we asked the following question (in German): 1. *Thinking back to the time since the beginning of the COVID-19 pandemic in March [2020]: What was, for you personally, the worst thing about the COVID-19 crisis? Please describe it in a few words in the space below.* 2. *And what was, for you personally, the best thing about the COVID-19 pandemic? Please describe it in a few words in the space below.* These questions were also asked in mid-September, but using the “*COVID-19 summer”* as the reference frame.

### Data analysis

Data were analyzed using thematic analysis. Thematic analysis allows the identification of key themes in large bodies of qualitative data. Because this was a novel research topic, an inductive (data-driven) bottom-up approach was used. Each answer was given equal attention in the coding process and was analyzed without a pre-existing framework or codebook. This approach allows for the identification of unanticipated insights, and, thus, the generation of new data-driven research questions in the future. We used a semantic approach; thus, only explicit meaning was coded, without interpreting the underlying meaning, reasons, or tone. Circumstances changed rapidly between May and September 2020; therefore, we analyzed the answers provided during each assessment separately. For a detailed description of the analytic process, see Braun and Clarke (2006).

Each answer was coded using the NVivo software. Given the large quantity of answers, three levels of abstraction – codes, subthemes, and themes (from specific to more generic) – were necessary to arrive at the final overarching sets of “best” and “worst” themes. First, we created codes based on participants’ answers. Subsequently, we summarized similar codes into larger subthemes. Similar subthemes were then classified into larger themes. Because the coding was inductive, no codebook with predefined themes was used.

We used the online Miro data visualization tool and mind maps within this tool to categorize codes into subthemes and themes. The Miro software emulates creating “sticky notes” of each code and grouping them together based on similarities. Thus, each code was given the same level of attention, regardless of the number of times it was coded. In the next stage, themes were compared to the original text to ensure that they were representative of the data. Because the survey was conducted in German, the analyses of the answers were conducted by two native German speakers. Themes, codes, and example quotes were then translated into English via back translation. Table 1a contains examples of how answers were coded, and then assigned to a subtheme and theme. Most codes were classified into a single theme; however, some codes were assigned to more than one theme (see Table 1b).

**Table 1:** Illustrations of how quotes were coded and then assigned to subthemes and themes

a) Examples of how answers were coded and then assigned to larger subthemes and overarching themes			
Quote ➔	Code ➔	Subtheme ➔	Theme
*“That I passed my training without having to sit an exam. An absolute highlight!”*	Passed semester/studies automatically	No exam stress	Positive changes in work and education
*“At the beginning and until mid-May all gyms were closed. Next to my studies I need exercise for balance, and without open gyms it is much harder to motivate myself to exercise as much.”*	Gyms closed and no team sports	Closed public spaces	Restrictions of freedom
*“That I couldn’t exercise my hobby.* *I could not play football with my football team.”*	Gyms closed and no team sports	Closed public spaces	Restrictions of freedom
“*No longer being able to go to restaurants and only seeing certain people.”*	Closing of bars and restaurants	Closed public spaces	Restrictions of freedom
b) Example of a participant’s answer that was subsequently included in two different codes			
Quote ➔	Codes		
*“The uncertainty. Sometimes I have the feeling that the problems we are faced with will change everything. With that I mean the climate crisis, macrobiotics developing resistance, political instabilities etc. There is a huge fear of the future tied to that, the feeling of maybe never being able to build a family or to find a job. The Corona crisis sometimes feels like a rolling stone.”*	Uncertainty (future) Worry		

Thematic analysis typically does not employ a positivist approach to data analysis (such as counting the number of answers represented in each theme). However, due to the large number of descriptions in our study (*N*=1,462), we report how many times a code was assigned and which themes were generated from the most common codes. This does not indicate that certain themes are more important than others, but it does indicate the frequency with which themes were coded in participants’ answers. A PhD student and a research assistant with a MSc degree and no previous experience in risk and resilience research conducted the thematic analysis. No codebook was used to train raters on “accurate” coding (Braun & Clarke, 2006). Rather, developing the codes was an organic process that evolved in the coding process. Therefore, we do not report inter-rater reliability for “accurate” coding.

## Results

### Demographic characteristics of respondents

Table 2 presents descriptive statistics. The majority of young adults in the analytic sample were female, lived in shared households, and had a medium level of education (i.e., they had completed vocational/compulsory education, were currently in education/training, or were employed). Consistent with the diverse Zurich population, 42% of participants were from families where both parents had come to Switzerland from abroad, including from Serbia/Kosovo, Germany, Sri Lanka, and Turkey, among other countries.

**Table 2:** Descriptive statistics for respondents who participated either in the late-May or in the mid-September z-proso COVID-19 survey or both (*N*=517)

	Mean	SD	*N*	%
Female			313	60.5
Age	22.5	0.36		
Family ISEI (range: 16-90)	53.9	19.3		
Migration background (1=both parents born abroad)			216	42.3
Education (age 20)				
Low (NEET)			8	1.5
Medium			323	62.5
High			186	36.0
Living alone			25	4.9

*Note*. ISEI: International Socioeconomic Index of occupational status; NEET: not in education, employment, or training. Age shown at the time of the Spring 2020 COVID-19 surveys

### The best about the lockdown: May 2020

**Figure 1:**
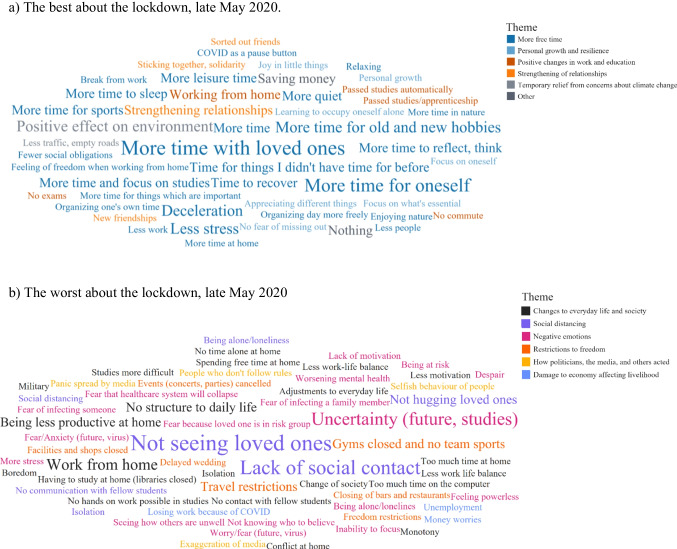
Word cloud with codes and frequency of coding. Color-coded by overarching theme. Larger font size indicates greater frequency

We follow the guidelines established by Braun and Clarke (2019) in reporting our results. Table 3a indicates all generated themes and subthemes as well as their frequency; Figure 1a presents a word cloud with codes and their frequency. Word clouds were created using tableau version 2020.4 and serve a visual illustration of our findings. They depict all codes that were coded more than once; the colors show the theme that codes were categorized into. A full list of individual codes associated with each subtheme can be found in the electronic appendix (Table S1a). Below, we describe the themes coded *n* ≥ 25 times and provide example quotes.

**Table 3:** Themes and subthemes that were coded as a) the best and b) the worst of the COVID-19 pandemic in late May 2020

(a) The best about the lockdown: themes, subthemes, and frequency of coding			
Themes	Times coded	Subthemes	Times coded
More free time	349	More time for one’s own needs	154
		Deceleration of life	70
		More time with others/loved ones	59
		More free time in general	37
		More space/time for oneself	15
		Gaining control of one’s own time	14
Personal growth and resilience	54	Resilience building	21
		New focus in life	13
		Gratitude/thankfulness for one’s own situation	10
		Appreciation for own country/the society in which we live	≤5
		Finding joy in the little things	≤5
Positive changes in work and education	41	Working from home	23
		No exam stress	9
		New opportunities	≤5
		Work-related positive changes	≤5
Strengthening of relationships	38	Strengthening of relationships	33
		Decisions about who should be part of one’s daily life	≤5
Temporary relief from concerns about climate change	26	Positive effect on nature/environment	26
Nothing	14	Nothing	14
Saving money	12	Saving money	12
Switzerland	≤5	Appreciating vacationing in and exploring Switzerland	
(b) The worst about the lockdown: themes, subthemes, and frequency of coding			
Themes	Times coded	Subthemes	Times coded
Changes to everyday life and society	129	Changes in terms of work/studies	44
		Changes in everyday life	29
		Less structure in daily life	26
		More bored during pandemic	14
		Less likely to be home alone	11
		Other subthemes: Being pregnant is more difficult during pandemic, changes to society, harder to find apartment	≤5
Social distancing	127	Social distancing	127
Negative emotions	110	Uncertainty in the present moment and regarding future	50
		Negative emotions (e.g., worry/fear, despair, hopelessness)	36
		Fear that loved ones may be infected/affected by COVID illness	18
		Worsening mental health	6
Restrictions to freedom	61	Closed public places	33
		Travel restrictions	15
		Restrictions of freedom	13
How politicians, media and others acted/handled the situation	30	Poor handling of situation by media and politicians (criticism)	15
		How others acted/did or did not comply with measures	9
		Disagreement, polarizations of opinions	6
Damage to economy affecting livelihood	19	Damage to economy affecting livelihood	19
Nothing	≤5	Nothing	≤5
People dying	≤5	That people are dying	≤5

### Best, theme 1: more free time (coded *n*=349 times)

For many participants, the best aspect of the lockdown was the deceleration of life (*n*=70, with n referring to the number of times this theme was coded), having more free time in general (*n*=37), and, in particular, having more time to fulfill one’s own needs (*n*=154). This included having more time for hobbies and sports, more time with loved ones, and more time to sleep, reflect, think, and relax/recover.

*“So much time for all the things that I have always wanted to do.”*

*“FINALLY having some time for myself, without the pressure or stress of having to do something else (other than university work).”*

### Best, theme 2: personal growth and resilience (coded *n*=54 times)

Personal growth and resilience were reported by many. This included focusing on the positive aspects of difficult situations, learning to handle crisis situations, being thankful, and appreciating what one has, where one lives, the people surrounding one, and health.

*“You learn to see the positive in difficult situations! And to learn from it.”*

*“Focusing on the really important things in life and maybe becoming more humble again.”*

### Best, theme 3: positive changes in work and education (coded *n*=41 times)

Another theme was positive changes in work and education. This included working from home (*n*=23) and successfully passing university/apprenticeship milestones without having to take (in-person) exams, meaning that exam stress was reduced or eliminated.

*“Working comfortably from home.”*

*“Exams were cancelled.”*

### Best, theme 4: strengthening of relationships (coded *n*=38 times)

Many participants reported strengthening relationships as the best aspect of lockdown. Forging new friendships, deepening existing ones, and social contact in general were codes associated with this theme. Interestingly, the lockdown was described as a time to reevaluate who should be part of one’s life; several participants reported sorting out friends, seeing through people’s facades, or severing ties with “fake” people as the best aspects of the lockdown.

*“A much stronger bond with my family.”*

*“I have realized how important some friendships are, and that some friends are not real friends at all.”*

### Best, theme 5: temporary relief from concerns about climate change (coded *n*=26 times)

Several participants expressed that the best aspect of the lockdown was its positive effect on the environment, including nature being able to “regenerate” and “recover” and the fact that fewer planes were flying and that city traffic was reduced.

*“Of course also that nature can finally breathe again.”*

In relation to the theme of more free time, nature was also mentioned repeatedly. Some respondents described spending more time in nature and enjoying nature as the best elements of lockdown. Others stated that they discovered hiking and explored Switzerland.

*“On top of this, I was able to enjoy nature where I live more by going on walks.”*

Additional best aspects of the pandemic themes that were coded *n* < 25 times included “nothing” (*n*=14), “saving money” (*n*=12), and “Switzerland” (i.e., exploring Switzerland; *n*≤5).

### The worst about the lockdown: May 2020

Table 3b indicates all generated themes and subthemes and the frequency of coding (see also Figure 1b). A full list, including individual codes associated with each subtheme can be found in the electronic appendix (Table S1b). Below, we describe the themes endorsed by *n* ≥ 25 and provide example quotes.

### Worst, theme 1: changes to everyday life and society (coded *n*=129 times)

For many young adults, changes to everyday life and society, including changes to work and daily structure, constituted the worst aspects of the Spring 2020 lockdown. Indeed, although the “best” themes indicated that many participants perceived positive elements associated with home office and education, others expressed that changes in their work and studies such as transition to working or studying remotely was among the worst aspect of the pandemic (*n*=44). Some participants reported that they were not able to have alone-time at home (*n*=11; i.e., always being surrounded by other people in the home bothered them), or that they suffered from boredom and difficulties concentrating. Indeed, many participants were unable to maintain a daily routine and lacked structure (*n*=26).

*“That I don’t have a daily rhythm anymore and that I have to study at home.”*

*“Being at home all the time.”*

Thus, while gaining control of one’s own time was reported as one of the best aspects of the pandemic by some, the change in daily structure was considered a challenge by others.

### Worst, theme 2: social distancing (coded *n*=127 times)

Social distancing was among the most difficult aspects of the pandemic for young adults. This encompassed codes of not seeing loved ones (*n*=51), and also a lack of physical contact (e.g., not hugging loved ones; *n*=10).

*“Not meeting up with friends and family.”*

*“Not being able to hug loved ones (grandparents, parents, siblings, close friends).”*

### Worst, theme 3: negative emotions (coded *n*=110 times)

Many participants highlighted uncertainty about the present and the future as the most difficult aspects of the pandemic (*n*=50).

*“Uncertainty about the whole situation, the change to studying from home, work and exams, the fear that something could happen to someone, the loss of social contact, etc.”*

Indeed, several participants reported negative emotions, such as despair, loneliness, fear, and powerlessness. The feeling of fear was frequently associated with COVID-19-related illness (*n*=18); for example, fearing that their loved ones would become infected or that they personally would infect a family member (e.g., grandparents).

*“Feeling alone.”*

*“The worry of falling ill and infecting people around me, mostly my parents, who are in the “at risk” group.”*

Notably, a corresponding theme of “positive emotions” was not coded for the best aspects of the pandemic.

### Worst, theme 4: restrictions to freedom (coded *n*=61 times)

Codes associated with the theme of restrictions to freedom (*n*=61) included bans on gatherings, closed public places (shops, bars, restaurants), and travel restrictions. Another associated code was the closing of gyms, and being unable to engage in team sports (*n*=16).

*“Not being able to do anything anymore.”*

*“Not being able to exercise as usual.”*

### Worst, theme 5: how politicians, the media, and others acted (coded *n*=30 times)

Several participants expressed that how other people handled the situation was the worst aspect of the pandemic. This included the dissemination of misinformation and fearmongering by politicians and the media (*n*=15), as well as others who did not comply with the imposed restrictions.

*“That the media presented everything sensationally and didn’t allow any peace.”*

Several respondents also referred to conspiracy theories and polarization of opinions (*n*=6):

*“That demonstrations happened and that some people who participated in them believe in conspiracy theories and are spreading them through different channels.”*

Additional worst aspects of the pandemic themes that were coded *n* < 25 times included “damage to the economy, affecting livelihood” (*n*=19), “nothing” (*n ≤* 5), and “that people are dying” (*n ≤* 5).

### The best and the worst of the first COVID summer: September 2020 assessment

We now briefly describe the primary findings from the Fall 2020 survey, which reflected back on summer 2020. Many themes overlap with the findings from late May 2020. We report on a few main themes, but mostly those that were newly coded in September 2020. For a full list of themes and subthemes, see electronic appendix, Table S2 and Figure S1).

### More time (coded *n*=203 times) and personal growth (coded *n*=57 times)

The theme of the deceleration of life was at the forefront again (*n*=65). Indeed, having more time for hobbies and personal needs (*n*=56), as well as more time with others (*n*=59), remained major themes. Strengthening relationships through solidarity, spending more time with family, personal growth, and building resilience became dominant themes; participants noted that they had more patience and more time for reflection and growth.

*“The lockdown also meant slowdown, time to reflect and space to mindfully review and come to terms with one’s personal situation and ambitions.”*

*“I used the time to reflect about my life and work. All in all, the Corona situation did me some good.”*

### Travel restrictions (coded *n*=73 times)

Many participants wrote that not being able to travel (to other countries) was the worst aspect of the summer lockdown. Some, however, also mentioned that their friends being nearby during the summer and spending additional time with them was the best aspect. A notable number of participants stated that the best element of the COVID-19 summer was spending holidays in Switzerland and exploring their own country (*n*=37).

*“The newly found time and that almost all my friends were in Zurich and not travelling abroad. Everyone was more available and therefore able to be more spontaneous.”*

*“I did not have the feeling of ‘oh this year I have to travel somewhere by plane.’ You can also have wonderful vacations in Switzerland.”*

### Always having to be careful (coded *n*=11 times)

Within the overarching theme of negative emotions, a new subtheme was coded: Wariness about always having to be careful. This included worrying about whether to go somewhere, how other people would perceive one if one did, and finding it stressful to meet people. In contrast, only one participant expressed this in the Spring 2020 survey.

*“Always having to be careful when too many people are gathered in one place.”*

## Discussion

Risk and resilience research has laid important foundations for and made substantial progress in understanding the development of psychopathology and better-than-expected adjustment in the face of risk (e.g., Masten, 2001; Rutter, 1990; Werner, 1993). Nevertheless, we have seen historic declines in young people’s mental health (Keyes et al., 2019; Twenge et al., 2019), and the repeated investigation of long-appreciated concepts and well-accepted measures (Luthar et al., 2021) has not generated the insights needed to reverse this trend.

During this status quo, the COVID-19 pandemic constituted an opportunity to uncover new knowledge for the science of risk and resilience (Roubinov et al., 2020). The first lockdown collectively paused and interrupted life. Indeed, it provided people with the opportunity to re(assess) their life. Surprisingly, between 19-35% of people reported feeling better in the early months of the pandemic than before (e.g., Luthar et al., 2021; Panzeri et al., 2021; Penner et al., 2021; Shanahan et al., 2022; Silk et al., 2021; Soneson et al., 2022). To investigate this pattern, we asked young adults directly to describe the best and the worst aspects of their early pandemic lives, with the aim of generating new insights into risk and protective processes.

### The deceleration of life and a new-found abundance of free time

For many young adults, having additional free time was the best aspect of the lockdown/pandemic, and positively contrasted with a perceived lack of time beforehand. The time necessary for work/education, commuting, consuming social and digital media, socializing, and completing mundane tasks (e.g., errands, doctor’s appointments) often exceeds young people’s time resources. Although structured activities can protect young people’s mental health (Mahoney & Vest, 2012), overscheduling, time pressures, and perceived lack of control over one’s time can induce stress, anxiety, and depression (e.g., Abeles, 2016; Brown et al., 2011; Luthar et al., 2020). Indeed, when time resources are scarce, healthy behaviors (e.g., exercise, sleep) are often reduced or abandoned, meaning that these reliable mood boosters no longer shield overscheduled young people from mental health problems.

The collective slow-down in Spring 2020 provided young adults with the unprecedented opportunity to relinquish activities and relationships that they did not enjoy. Furthermore, their “fear-of-missing-out” (FOMO) on exciting events (e.g., Oberst et al., 2017) was eliminated. Many young people used the additional time to improve their self-knowledge and to pursue rewarding and resilience-building activities. These included newly (re)discovered hobbies and time spent in meaningful relationships, in nature, and engaging in healthy behaviors. Previous quantitative work also suggested that time devoted to hobbies, healthy behaviors, and nature, and identifying positive aspects of the situation were correlated with positive during-pandemic well-being (e.g., Lades et al., 2020; Shanahan et al., 2022; Silk et al., 2021).

Perhaps, the newfound abundance of free time emerged as the best aspect of the pandemic simply because other positive aspects were lacking. Indeed, even the additional free time was also not enjoyed by all, ranking among the worst aspects of the pandemic for some. Those who had previously achieved a balanced time budget may have struggled with the disruption of their routine. Such struggles may have been more prominent for extraverted young adults (Wijngaards et al., 2020) and those living alone (Steinhoff et al., 2021) – who may have felt particularly isolated, lonely, and bored during the lockdown. Furthermore, although FOMO with respect to current events was reduced (see also, Elmer et al., 2020), it intensified with respect to the attainment of developmental milestones that typically constitute a successful transition to adulthood.

*Implications:* Risk and resilience research needs to assess young people’s perceived time pressures, overscheduling, and signs of burnout (e.g., Tuominen-Soini & Salmela-Aro, 2014). The sociological literature on “role strain” has examined the difficulties of balancing multiple commitments and obligations in middle adulthood (e.g., Goode, 1960); such time and role strains have also become a prominent challenge of young adulthood.

Overscheduled young adults may benefit from scheduled free time, or mini-sabbaticals, during non-pandemic times to improve their self-knowledge and personal development, to reflect on their life and their values, and to pursue rewarding and resilience-building activities (e.g., Ceary et al., 2019). Occasionally “hitting the pause button” may also allow young people to (re)discover the simple pleasures of life that sometimes get lost in the overstimulation of the digital era. Because FOMO is a threat to mental health (e.g., Oberst et al., 2017), scheduling free time collectively, or at the institutional level (e.g., in college, in young adults’ workplaces), may be particularly beneficial. It should not take a global pandemic for young people to find time and space for personal growth.

### Work and educational pressures

Young adulthood is a significant period of educational and professional development and is often accompanied by heavy workloads and time commitments, high-stakes testing, and stressful new job market experiences (e.g., Arnett, 2000); many young people suffer from these pressures (e.g., Luthar et al., 2020; Luthar et al., 2021). Before the pandemic, high-stakes educational testing was linked with mental distress and self-injury in this sample (Steinhoff et al., 2020). The reduction of work and educational pressures during the pandemic, and having more time to take breaks and recover while working or studying remotely was a positive aspect for many. Physical distance also came with the elimination of in-person stressors such as bullying, competitive work/educational situations, and unfavorable social comparisons, which likely contributed to a relief from pressures (e.g., Luthar et al., 2020; Silk et al., 2021).

Work and education also involve many enjoyable aspects, which young adults missed during the lockdown. For example, they reported missing the energizing and motivating aspects of in-person contact, the technical infrastructure necessary to complete tasks efficiently, and the separation of work and life, which was often not possible in their home office setup.

*Implications:* Risk and resilience research needs to routinely assess risk and protective factors related to young people’s educational and workplace settings. These are important to understand, given also the “great resignation” (or quitting work) trend that has affected some (young) workers in Western labor forces (Ksinan Jiskrova, 2022), and which may be due, in part, to the mental health costs and pressures of jobs that are not sustainable in the long-term. In turn, positive workplace social interactions and meaningful work can promote mental health.

### Disruption, change, and uncertainty

Disruptions due to the COVID-19 pandemic and uncertainty were among the strongest correlates of emotional distress in previous quantitative work; this was further illuminated by the qualitative data. Young adults were especially concerned about the disruptions to routines and uncertainties related to their educational and professional futures. In addition, they worried about potential illness, and the future (personal, societal, global), including disruptions to their life plans. Indeed, several participants noted that the increased difficulty of achieving the expected milestones of young adulthood (e.g., interviewing for jobs, changing jobs, getting married, becoming a parent) was the worst aspect of the pandemic for them.

Difficulties with tolerating uncertainty can be fertile grounds for intensifying anxiety and depression (e.g., Carleton et al., 2012; Peters et al., 2017). Indeed, a recently updated definition of stress emphasizes uncertainty as a key component: stress is *“… the individual state of uncertainty about what needs to be done to safeguard physical, mental or social wellbeing”* (p. 184, Peters et al., 2017). Yet, uncertainty and rapid change may be a constant of current times.

*Implications:* Young people’s flexibility and skills to cope with uncertainty and change may be key mechanisms to increase resilience (e.g., Birrell et al., 2011). Our September 2020 results supported this, with some young adults reporting an increased sense of agency and self-efficacy after successfully having mastered novel challenges during the pandemic.

### Climate change

Concerns about the climate crisis constitute significant psychological stressors, but remain understudied (Doherty & Clayton, 2011). Similar to economic stressors, climate crisis concerns/ecoanxiety constitute chronic existential fears (e.g., Dodds, 2021). Individuals’ control over climate change is limited. Furthermore, concerns about climate change are often accompanied by disappointment in other people’s or society’s (lack of) actions, possibly compounding stress. As the climate crisis worsens, such concerns will likely amplify young people’s resignation and hopelessness (Fritze et al., 2008). In turn, our findings suggest that measures to slow or pause climate change (e.g., reduction in global travel and city traffic during lockdowns) enhance hope and well-being among climate-concerned young adults.

*Implications:* Climate crisis-related stressors are significant concerns that warrant inclusion in future risk and resilience research. Organizing young people to engage in constructive, concrete steps to combat climate change may benefit their mental health.

### Divisions in society, fatigue from restrictions, cautions, and chronic pandemic-stress

Many of the reported best and worst aspects of the pandemic were the same in Spring and Fall 2020: more free time was among the best aspects, and the imposed restrictions and social distancing were among the worst aspects at each assessment. In the Spring, some participants had initially mentioned solidarity and “sticking together” as the best aspects. Yet, beginning in the Spring, and even moreso in the Fall of 2020, participants increasingly reported other people’s reactions to the pandemic – including pandemic-related behaviors, attitudes, and beliefs, the division and polarization of opinions, and losing common ground with others – as the worst aspects of the pandemic.

The Fall 2020 responses also highlighted the increasing chronicity and wear and tear from pandemic-related stressors. For example, young adults reported being fatigued from having to comply with ever-changing restrictions and continuously having to be cautious. These findings are consistent with reports that mental health problems became more common later in the pandemic (Racine et al., 2021).

*Implications:* Perceived cohesion/solidarity versus division of society are understudied factors that likely influence young people’s well-being. Such factors may be especially salient during times of social change, suggesting a source of risk originating at the societal level. The changing nature and increasing chronicity of (pandemic-related) stressors needs to be captured in empirical risk and resilience research.

### Screen time and social and digital media

Increases in social and digital media use have coincided with declines in young people’s mental health (e.g., Twenge et al., 2020). Yet, social media use does not exert a uniformly negative influence (Odgers & Jensen, 2020). In the current study, young adults did not mention social and digital media as the best or the worst aspects of the pandemic. An exception was the humor of COVID-memes, which was reported to be the best aspect of the pandemic by a few.

Why were social and digital media not mentioned as a negative aspect? First, social media was often the only means for maintaining social contact during physically distanced times. Combined with our findings that people primarily focused on positive social relationships during the lockdown, while abandoning negative ones, social media engagement could have (temporarily) lost some of its potentially negative effects (e.g., Gadassi Polack et al., 2021). Second, during the lockdown, venues for gatherings and parties (e.g., restaurants, clubs, concerts) closed. Thus, FOMO-induced stress as a negative aspect of social media (e.g., Oberst et al., 2017) was temporarily eliminated. Third, although the absolute amount of time spent on social media likely increased during the pandemic, it may have detracted less from engaging in healthy or other enjoyable activities than during non-pandemic times – given the greater abundance of free time during the pandemic.

*Implications:* The impact of interacting with social and digital media on young people’s well-being is varied. Not a single young adult reported social media as the worst aspect of the pandemic in our study. Social media involvement needs to be more routinely assessed to understand its impact on risk and resilience in different contexts.

### Interim summary and implications

While exposed to a historic novel stressor, and amidst a youth mental health crisis, young adults had the chance to (re)assess their lives. Insights into their well-being during the pandemic can inform future risk and resilience research. Emerging themes included time pressures and educational/work pressures that take away from free time for hobbies, important others, self-development, health behaviors, and the development of resilience skills. Themes also revolved around climate change concerns, divisions in society, the global future, and difficulties adjusting to disruptions, uncertainty, and change. These themes highlight that many of the stressors faced by young adults involve systemic structural and societal factors that young adults have limited control over. Society and organizations, including work places and schools, must contribute to changes that reduce risk and increase mental health resilience in young people.

From a methodological point of view, our findings support the need for statistical models that capture the complexity of risk and resilience processes. For example, more free time was the best aspect of the pandemic for some young adults, but the worst for others. Measurement and analytic strategies must capture such heterogeneity in response to stressors. Many factors could have further modified the effect of more free time on well-being, including living arrangements, family and work situations, and socioeconomic status (e.g., Elmer et al., 2020; Steinhoff et al., 2021). Variable-centered analytic approaches that link population-level averages to one another often fail to capture such important nuances. Person-centered approaches with the capacity to model complex, multi-dimensional, dynamic interactions are key to understanding risk and resilience (e.g., Bergman & Magnusson, 1997; Muthen & Muthen, 2000).

An important next step in research includes theoretical thinking about the potential underlying causal mechanisms that link the factors identified here with well-being. Furthermore, research should measure the newly identified potential risk and protective factors and their putative causal mechanisms in quantitative research and examine which of the protective factors identified here optimally shield (which) young adults from stress, depression and anxiety, and other conditions underlying the youth mental health crisis. Ideally, the speed of the scientific process in the field of risk and resilience – including its iterations of getting input from young people, to developing theories and relevant measures, and testing them – needs to be increased. This may require the field to embrace qualitative data, and the methods to fully harvest them, more than before.

### Constraints on generalizability and additional limitations

Our study analyzed *N*=1,462 responses that described young adults’ best and worst experiences with the COVID-19 pandemic; it also came with limitations. First, as with most longitudinal studies, there was some attrition across assessments of the z-proso study and its COVID-19 surveys. Females and those with a Swiss background were more likely than males and those with a migrant background to participate in the COVID surveys; a small number of respondents skipped the open-ended section (which was placed at the end of the survey). Second, inductive thematic analysis is not a method designed to rank themes in their importance or to explain the reasons for why participants mentioned them. Third, our study exclusively examined young adults. Much previous risk and resilience research has focused on children or adolescents, and the best and worst aspects of the pandemic likely differ for these age groups.

Fourth, findings may not generalize to other countries. Switzerland underwent a lockdown, but did not enforce stay-at-home measures with law enforcement and also did not implement curfews. At the time, it also did not struggle with other acute political crises (e.g., social unrest, police-based violence) which could have further increased young people’s vulnerability to hopelessness and mental health problems. Furthermore, the health system in Zurich was not overwhelmed during our study period. The lack of extreme measures, other societal crises, or overburdening of the health care system may explain, at least in part, why the pandemic was not a uniformly negative experience for young people.

Fifth, the COVID-19 pandemic was an ever-changing stressor, and risk and resilience processes are indeed dynamic (Masten et al., 2021). Although we captured pandemic life at two different time points, risk and protective processes likely shifted as the pandemic lasted many additional months. Sixth, findings may not generalize to other types of crises that are characterized by different sets of stressors, such as wars, and the many traumas associated with them. Undoubtedly, each macro-level stressor has unique implications for risk and resilience. Nevertheless, we did identify factors that may generalize to other settings of stress. Indeed, most participants provided answers that compared and contrasted their during-pandemic life with their pre-pandemic life (e.g., having more time for hobbies during the pandemic than before). Seventh, the risk and protective factors discussed here may be specific to some outcomes (e.g., internalizing problems), and may not generalize to others (e.g., externalizing problems).

Finally, although inductive thematic analysis was well-suited for identifying novel themes for risk and resilience research, it was beyond the scope of this paper to quantify 1) the association of the newly identified themes with quantitative measures of mental health, 2) the relative importance of the themes to mental health, and 3) how participants’ answers to the open-ended questions differed by their sociodemographic characteristics or prior levels of stress and mental health. With new themes having been identified, we encourage addressing these questions in future quantitative research.

## Conclusion

The field of risk and resilience research has made tremendous progress through its various waves and iterations, including by leveraging natural and man-made disasters (e.g., hurricanes, ice-storms, mining-accidents) as scientific opportunities to gain new insights (e.g., Masten et al., 2021). Our study illustrates the value of going beyond traditionally studied risk and protective factors and methodological approaches to examine the more proximal factors in young peoples' lives that contribute to risk and resilience. Our approach allowed us to illuminate a macro-level exposure – like the COVID-19 pandemic – and its complex, both positive and negative, impact on young people, and to delineate specific features of these positive and negative experiences that can inform future research and interventions. The field of risk and resilience would benefit from greater use of approaches like qualitative interviewing and ecological momentary assessment that generate insights into more proximal experiences and mechanisms. The COVID-19 pandemic provided a unique opportunity to learn new lessons about the factors that influence young people’s mental well-being. We can “build back better” in risk and resilience research by going beyond long-appreciated concepts and well-accepted measures and by gleaning knowledge from young people directly about risk and protective factors in their mental health development that can then be tested in quantitative research.

## Supplementary materials

ESM 1 (PDF 2.35 MB)
