# Supplementary material for: The worst and the best: new insights into risk and resilience in young adults from the COVID-19 pandemic
Source: Advers Resil Sci. 2023 Apr 27;4(3):291–305. doi: 10.1007/s42844-023-00096-y (PMC10132952; doi:10.1007/s42844-023-00096-y)
Supplement: Supplementary file 1 — ESM 1 (PDF 2.35 MB) [file 42844_2023_96_MOESM1_ESM.docx]

**Online Supplement:**

**The Worst and the Best: New Insights into Risk and Resilience in Young Adults
from the COVID-19 Pandemic**

Shanahan, L., Johnson-Ferguson, L., Loher, M., Steinhoff, A., Bechtiger, L. Murray, A. J., Hepp, U., Ribeaud, D., & Eisner, M. (2023). *Adversity and Resilience Science.*

**Table S1**. Themes, subthemes, and codes that were coded as a) the best and b) the worst of the COVID-19 pandemic in late May 2020.

**Table S2**. Themes, subthemes, and codes that were coded as a) the best and b) the worst of the COVID-19 pandemic in September 2020.

**Figure S1.** Word cloud with codes and frequency of coding, September 2020.

*Content of Supplement starts on next page.*

**Table S1**. Themes, subthemes, and codes that were coded as a) the best and b) the worst of the COVID-19 pandemic in late May 2020.

**a.** The best about the lockdown (late May 2020):

| Themes | Times coded | Subthemes | Times coded | Codes Contained in Subtheme & Theme |
| --- | --- | --- | --- | --- |
| More free time | 349 | **More time for one’s own needs** | 154 | More time for oneself |
|  |  |  |  | More time for old and new hobbies |
|  |  |  |  | More time for sports |
|  |  |  |  | More time to reflect, think |
|  |  |  |  | More time and focus on studies |
|  |  |  |  | More time to sleep |
|  |  |  |  | More time at home |
|  |  |  |  | More time in nature |
|  |  |  |  | Time to recover |
|  |  |  |  | More time for things which are important |
|  |  |  |  | Enjoying nature |
|  |  |  |  | Animal crossing |
|  |  |  |  | More time to work on oneself |
|  |  |  |  | More time for things one enjoys |
|  |  |  |  | More time for nice things |
|  |  |  |  | More time to plan the future |
|  |  |  |  | Time to get a grip on life |
|  |  |  |  | Time to reorient |
|  |  |  |  | Time to develop/progress |
|  |  |  |  | Discovered hiking |
|  |  |  |  | Enjoying nice weather |
|  |  | **Deceleration of life** | 70 | Less stress |
|  |  |  |  | Deceleration |
|  |  |  |  | More quiet |
|  |  |  |  | Time to recover |
|  |  |  |  | Relaxing |
|  |  |  |  | Break from work |
|  |  |  |  | Less work |
|  |  |  |  | COVID as a pause button |
|  |  |  |  | Doing nothing |
|  |  |  |  | More mindfulness |
|  |  | **More time with others/loved ones** | 59 | More time with loved ones |
|  |  |  |  | More time with cat |
|  |  |  |  | Friends have more time |
|  |  |  |  | Dungeon and Dragons with friends |
|  |  | **More free time in general** | 37 | Time for things I didn't have time for before |
|  |  |  |  | More leisure time |
|  |  |  |  | More time |
|  |  | **More space/time for oneself** | 15 | Fewer people |
|  |  |  |  | Fewer social obligations |
|  |  |  |  | No stressful social encounters |
|  |  |  |  | Space from people |
|  |  |  |  | Didn't have to go where there are crowds of people |
|  |  | **Gaining control of one’s own time** | 14 | Organizing day more freely |
|  |  |  |  | Organizing one's own time |
|  |  |  |  | Feeling of freedom when working from home |
|  |  |  |  | Freedom to pursue other interests |
| Personal growth and resilience | 54 | **Resilience building** | 21 | Learning to occupy oneself alone |
|  |  |  |  | Personal growth |
|  |  |  |  | Better setting of priorities |
|  |  |  |  | Being more mindful about one's time |
|  |  |  |  | Learning to live day by day |
|  |  |  |  | Going on walks alone |
|  |  |  |  | Learning to see the positive things in difficult situations |
|  |  |  |  | Learning what is important and what makes you happy |
|  |  |  |  | Learning to deal with boredom |
|  |  |  |  | Getting to know and like oneself |
|  |  |  |  | Closeness to God |
|  |  |  |  | Learning to handle crisis situations |
|  |  |  |  | No guilt about being unproductive |
|  |  |  |  | Time to work on oneself |
|  |  |  |  | No fear of missing out |
|  |  | **New focus in life** | 13 | Focus on oneself |
|  |  |  |  | Focus on what's essential |
|  |  |  |  | Physical appearance no longer important |
|  |  |  |  | Getting important things done |
|  |  |  |  | Finding back to a new consciousness/focus |
|  |  | **Gratitude/thankfulness for one’s own situation** | 10 | Appreciating different things |
|  |  |  |  | Appreciating what we have |
|  |  |  |  | Being thankful that family is well |
|  |  |  |  | Appreciating normal life |
|  |  | **Appreciation for own country/society in which we live** | ≤5 | Being thankful for living in Switzerland |
|  |  |  |  | Appreciating living in Switzerland |
|  |  |  |  | Sticking together of people in Switzerland |
|  |  |  |  | Raising awareness in society about illness |
|  |  |  |  | The service provided by hospitals |
|  |  | **Finding joy in the little things** | ≤5 | Joy in little things |
|  |  |  |  | Being happier with less |
| Positive changes in work and education | 41 | **Working from home** | 23 | Working from home |
|  |  |  |  | No commute |
|  |  |  |  | Lasting changes like working from home |
|  |  | **No exam stress** | 9 | No exams |
|  |  |  |  | Passed studies/apprenticeship |
|  |  |  |  | Passed studies automatically |
|  |  | **New opportunities** | ≤5 | New opportunities |
|  |  |  |  | Paid furlough work |
|  |  |  |  | Innovation |
|  |  |  |  | Using skype creatively |
|  |  |  |  | New work |
|  |  | **Work related positive changes** | ≤5 | Working more efficiently |
|  |  |  |  | Routine |
|  |  |  |  | Working more |
|  |  |  |  | Continuing studies |
| Strengthening of relationships | 38 | **Strengthening of relationships** | 33 | Strengthening relationships |
|  |  |  |  | Sticking together, solidarity |
|  |  |  |  | New friendships |
|  |  |  |  | Contact with others |
|  |  |  |  | More video calls with friends |
|  |  |  |  | Realizing, how important people and social contact are |
|  |  |  |  | Attached importance to relationship |
|  |  |  |  | Charity/love for others |
|  |  |  |  | Social contacts |
|  |  |  |  | Conversations |
|  |  |  |  | Support from family |
|  |  | **Decisions about who should be part of one’s daily life** | ≤5 | Sorted out friends |
|  |  |  |  | Seeing the true face of people |
|  |  |  |  | People were more authentic |
|  |  |  |  | Fake people were cut out |
| Temporary relief from concerns about climate change | 26 | **Positive effect on nature** | 26 | Positive effect on environment |
|  |  |  |  | Less traffic, empty roads |
|  |  |  |  | No noise from planes |
| Other themes | 28 |  |  |  |
| Nothing | 14 | **Nothing** | 14 | Nothing |
| Saving   money | 12 | **Saving money** | 12 | Saving money |
| Switzerland | ≤5 | **Appreciating vacation in own country, discovery/exploring Switzerland** |  | Vacation in Switzerland |
|  |  |  |  | Exploring Switzerland |
| Individual codes | ≤5 |  |  | Reopening of shops |
|  |  |  |  | Everything |
|  |  |  |  | No alcohol |
|  |  |  |  | People finally learned to wash their hands |

**b.** The worst about the lockdown (late May 2020):

| Themes | Times coded | Subthemes | Times coded | Codes Contained in Subtheme & Theme |
| --- | --- | --- | --- | --- |
| Changes to everyday life and society | 129 | **Changes in terms of work/studies** | 44 | Work from home |
|  |  |  |  | Studies more difficult |
|  |  |  |  | Less work life balance |
|  |  |  |  | No contact with fellow students |
|  |  |  |  | Military |
|  |  |  |  | No hands-on work possible in studies |
|  |  |  |  | Changes at workplace |
|  |  |  |  | Internship cancelled |
|  |  |  |  | Education interrupted |
|  |  |  |  | Exams postponed |
|  |  |  |  | Couldn't complete final exam |
|  |  | **Changes in everyday life** | 29 | Too much time at home |
|  |  |  |  | Isolation |
|  |  |  |  | Having to study at home (libraries closed) |
|  |  |  |  | Adjustments to everyday life |
|  |  |  |  | Spending free time at home |
|  |  |  |  | Not being able to go home |
|  |  |  |  | Not having used time efficiently |
|  |  |  |  | Not attaining goals and wishes |
|  |  |  |  | Household infected with COVID |
|  |  |  |  | Less leisure time |
|  |  | **Less structure in daily life** | 26 | No structure to daily life |
|  |  |  |  | Being less productive at home |
|  |  |  |  | Less work-life balance |
|  |  |  |  | Too much time on the computer |
|  |  | **More bored during pandemic** | 14 | Monotony |
|  |  |  |  | Boredom |
|  |  |  |  | Less motivation |
|  |  |  |  | Alcohol use because of boredom |
|  |  |  |  | Nothing to fill free time |
|  |  | **Less likely to be home alone during pandemic** | 11 | Conflict at home |
|  |  |  |  | No time alone at home |
|  |  |  |  | Not being able to be alone |
|  |  |  |  | Not being able to learn in peace |
|  |  | **Being pregnant is more difficult during pandemic** | ≤5 | Difficult pregnancy |
|  |  |  |  | No being able to enjoy pregnancy |
|  |  | **Changes to society** | ≤5 | Change of society |
|  |  | **Harder to find apartment** | ≤5 | Harder to find apartment |
| Social distancing | 127 | **Social distancing** | 127 | Not seeing loved ones |
|  |  |  |  | Lack of social contact |
|  |  |  |  | Not hugging loved ones |
|  |  |  |  | Being alone/loneliness |
|  |  |  |  | Isolation |
|  |  |  |  | Social distancing |
|  |  |  |  | No communication with fellow students |
|  |  |  |  | No normal time spent with loved ones |
| Negative emotions | 110 | **Uncertainty in the moment (present) and regarding future** | 50 | Uncertainty (future, studies) |
|  |  |  |  | Fear/Anxiety (future, virus) |
|  |  |  |  | Fear that healthcare system will collapse |
|  |  |  |  | Being at risk |
|  |  |  |  | Not knowing who to believe |
|  |  |  |  | Lack of clarity about COVID |
|  |  |  |  | Fear that parents will become unemployed |
|  |  |  |  | Uncertainty about visa |
|  |  |  |  | Inability to plan |
|  |  |  |  | Future plans ruined |
|  |  |  |  | Future will be tougher for youth |
|  |  |  |  | Uncertainty about employment |
|  |  | **Negative emotions** | 36 | Being alone/loneliness |
|  |  |  |  | Worry/fear (Future, virus) |
|  |  |  |  | Seeing how others are unwell |
|  |  |  |  | Lack of motivation |
|  |  |  |  | Inability to focus |
|  |  |  |  | Feeling powerless |
|  |  |  |  | Despair |
|  |  |  |  | The feeling of not being able to keep up |
|  |  |  |  | Being overwhelmed |
|  |  |  |  | Hopelessness |
|  |  |  |  | Lack of meaning |
|  |  |  |  | Ruminating |
|  |  |  |  | Mistrust |
|  |  |  |  | Having to always wonder if something (i.e., actions) are ok or not |
|  |  | **Fear that loved ones may be infected/affected by COVID illness** | 18 | Fear of infecting a family member |
|  |  |  |  | Fear of infecting someone |
|  |  |  |  | Fear because loved one is in risk group |
|  |  |  |  | Worry about grandparents |
|  |  | **Worsening mental health** | 6 | Worsening mental health |
|  |  |  |  | More stress |
|  |  |  |  | No distraction from depression and anxiety |
| Restrictions of freedom | 61 | **Closed public places** | 33 | Gyms closed and no team sports |
|  |  |  |  | Closing of bars and restaurants |
|  |  |  |  | Events (concerts, parties) cancelled |
|  |  |  |  | Facilities and shops closed |
|  |  |  |  | Opening times |
|  |  |  |  | Ban on gatherings |
|  |  |  |  | Unable to go to funeral |
|  |  | **Travel restrictions** | 15 | Travel restrictions |
|  |  | **Restrictions of freedom** | 13 | Freedom restrictions |
|  |  |  |  | Delayed wedding |
|  |  |  |  | Not being able to do anything anymore |
|  |  |  |  | Nothing to do during free time |
|  |  |  |  | Not being allowed outside |
|  |  |  |  | Having to wear masks |
|  |  |  |  | Not being as active as usual |
|  |  |  |  | Hobbies no longer possible |
| How politicians, media and others acted/ handled the situation | 30 | **Poor handling of situation by media and politicians (criticism)** | 15 | Panic spread by media |
|  |  |  |  | Exaggeration by media |
|  |  |  |  | Poor communication between scientists and media |
|  |  |  |  | Lies of politicians |
|  |  |  |  | False information from the media |
|  |  |  |  | Switzerland acted too late |
|  |  |  |  | Situation in the USA |
|  |  | **How others acted/did or did not comply with measures** | 9 | People who don't follow rules |
|  |  |  |  | Selfish behavior of people |
|  |  |  |  | Little solidarity |
|  |  |  |  | Many don't take it seriously |
|  |  |  |  | Others spreading misinformation |
|  |  |  |  | Irresponsibility |
|  |  | **Disagreement, polarizations of opinions** | 6 | Conspiracy theories |
|  |  |  |  | That people let themselves be steered by fear without thinking |
|  |  |  |  | Stupidity of people |
|  |  |  |  | Realizing that COVID is not dangerous and that I was manipulated |
|  |  |  |  | Being told off for not following the 2-meter distancing rule |
| Damage to economy affecting livelihood | 19 | **Damage to economy affecting livelihood** | 19 | Money worries |
|  |  |  |  | Unemployment |
|  |  |  |  | Losing work because of COVID |
|  |  |  |  | Difficulty finding work |
|  |  |  |  | Job opportunity lost because of COVID |
|  |  |  |  | Not being able to change work |
| Other themes |  |  |  |  |
| Nothing | ≤5 |  | ≤5 | Nothing |
| People   are dying | ≤5 |  | ≤5 | That people are dying |

**Table S2**. Themes, subthemes, and codes that were coded as a) the best and b) the worst of the COVID-19 pandemic in September 2020.

**a***.* The best about the COVID-19 summer: themes, subthemes, and codes included.

| **Themes** | **Times coded** | **Subthemes** | **Times coded** | **Codes Contained in Subtheme & Theme** |
| --- | --- | --- | --- | --- |
| **More free time** | 203 | **Deceleration of life** | 65 | Fewer people and crowds |
|  |  |  |  | Deceleration, relaxing, recovery |
|  |  |  |  | No obligations |
|  |  |  |  | Quiet |
|  |  |  |  | Less stress |
|  |  |  |  | No travel stress |
|  |  |  |  | Sleeping in |
|  |  |  |  | Switching off |
|  |  |  |  | Less consumption |
|  |  |  | 59 | More time with loved ones |
|  |  | **More time with others/loved ones** |  |  |
|  |  |  |  | Time with roommate |
|  |  | **More time for one’s own needs** | 56 | More time for oneself |
|  |  |  |  | More time for sport |
|  |  |  |  | More time for old and new hobbies |
|  |  |  |  | More time in nature |
|  |  |  |  | More time at home |
|  |  |  |  | Spending time outside |
|  |  |  |  | More time for studies |
|  |  |  |  | More time for personal projects |
|  |  |  |  | Beer pong |
|  |  |  |  | Time abroad |
|  |  |  |  | More time for political engagement |
|  |  | **More free time in general** | 19 | More leisure time |
|  |  |  |  | Time for things one didn't have before |
|  |  |  |  | More time |
|  |  | **Gaining control of one’s own time** | ≤5 | Gaining control over one's own time |
|  |  |  |  | Better time management than before COVID |
| **(Appreciation of) summer vacation** | 85 | **(Appreciation of) summer vacation** | 48 | Vacation |
|  |  |  |  | Good weather |
|  |  |  |  | Could go on vacation anyway |
|  |  |  |  | Vacation abroad |
|  |  |  |  | Going swimming |
|  |  |  |  | Spending time outside |
|  |  |  |  | Enjoying vacation |
|  |  |  |  | Vacation with family |
|  |  |  |  | Could enjoy the summer |
|  |  | **Appreciating vacation in own country, discovery/ exploring Switzerland** | 37 | Vacation and travel in Switzerland |
|  |  |  |  | Discovering Switzerland |
|  |  |  |  | Summer in Switzerland |
|  |  |  |  | Hiking in Switzerland |
|  |  |  |  | Vacation in the mountains |
|  |  |  |  | Enjoying Switzerland |
|  |  |  |  | People realize how beautiful Switzerland is |
| **Personal growth and resilience** | 57 | **Resilience building** | 27 | Time for personal growth |
|  |  |  |  | Self-reflection |
|  |  |  |  | Learning how to handle fears and thoughts |
|  |  |  |  | More patience |
|  |  |  |  | Made it through an episode of depression |
|  |  |  |  | Made the best out of the situation |
|  |  |  |  | Much creativity |
|  |  |  |  | Could focus more on nutrition |
|  |  | **New focus in life** | 15 | No fear of missing out |
|  |  |  |  | Focus on oneself |
|  |  |  |  | Focus on the important things |
|  |  |  |  | New opportunities |
|  |  |  |  | Everything is different |
|  |  |  |  | Fewer expenditures |
|  |  | **Gratitude/thankfulness for one’s own situation** | 11 | Being appreciative of/thankful for various things |
|  |  |  |  | Being thankful of doing well despite COVID |
|  |  |  |  | Staying healthy |
|  |  | **Finding joy in the little things** | ≤5 | Finding happiness in the little things |
|  |  |  |  | Happiness about things that are different because of COVID |
|  |  |  |  | Nice experiences |
|  |  |  |  | COVID memes, humor |
| **Positive changes in work and education** | 40 | **Positive changes in work and education** | 21 | New work |
|  |  |  |  | Keeping a routine |
|  |  |  |  | Work |
|  |  |  |  | Start of apprenticeship |
|  |  |  |  | Wrote bachelor/undergraduate thesis |
|  |  |  |  | Salary in spite of COVID |
|  |  |  |  | Quitting work |
|  |  |  |  | New work place |
|  |  |  |  | Start of studies |
|  |  |  |  | Found work through COVID |
|  |  |  |  | Studies not impacted negatively |
|  |  |  |  | Short work contract |
|  |  | **No exam stress** | 10 | Succeeded in studies without having to take an exam |
|  |  |  |  | Succeeded in studies |
|  |  | **Working from home** | 9 | Working from home |
|  |  |  |  | No commute |
|  |  |  |  | Could be at one place for longer |
| **Strengthening of relationships** | 38 | **Strengthening of relationships** | 38 | Friends were there because they could not go abroad |
|  |  |  |  | Strengthening of relationships |
|  |  |  |  | New relationships |
|  |  |  |  | More contact with family |
|  |  |  |  | Solidarity/sticking together |
|  |  |  |  | Partner moved to Switzerland |
|  |  |  |  | Getting to know new people |
|  |  |  |  | Being able to see family anyway |
|  |  |  |  | Keeping contact with close friends |
|  |  |  |  | No fighting/conflict with anyone |
|  |  |  |  | Realizing who is important |
| **Accomplishment of normative social milestones** | 13 | **Accomplishment of normative social milestones** | 13 | New apartment |
|  |  |  |  | Moving out of parents’ home |
|  |  |  |  | Got engaged |
|  |  |  |  | Fell in love |
|  |  |  |  | Pregnancy |
| **Regaining freedom** | 9 | **Regaining freedom** | 9 | Loosening of restrictions |
|  |  |  |  | No complete lockdown |
| **Temporary relief from concerns about climate change** | 7 | **Positive effect on nature** | 7 | Impact on environment |
|  |  |  |  |  |
| **Other themes** | 46 |  |  |  |
| **Nothing** | 36 | **Nothing** | 36 | Nothing |
| **Saving money** | 10 | **Saving money** | 10 | Saving money |
|  |  |  |  |  |
| **Individual codes** | 6 |  |  |  |
|  |  |  |  | Summer is over |
|  |  |  |  | Switzerland handled the crisis well |
|  |  |  |  | Excuse to cancel plans |
|  |  |  |  | Going to bars anyway |
|  |  |  |  | Quarantine during the military service |
|  |  |  |  | Feeling that COVID situation has stabilized |

**b***.* The worst about the COVID-19 summer: themes, subthemes, and codes included.

| Themes | Times coded | Subthemes | Times coded | Codes Contained in Subtheme & Theme |
| --- | --- | --- | --- | --- |
| Restrictions to freedom  (due to pandemic) | 183 | **Tr**  **Travel restrictions** | 73 | Travel restrictions |
|  |  | **Restrictions of freedom** | 39 | Obligation to wear masks |
|  |  |  |  | Freedom restrictions |
|  |  |  |  | Restricted freedom of movement |
|  |  |  |  | Restrictions |
|  |  |  |  | Lockdown |
|  |  |  |  | Not being able to do much |
|  |  |  |  | Couldn't do much with friends |
|  |  |  |  | Less spontaneity |
|  |  |  |  | Fine from police |
|  |  | **Impact on vacation** | 37 | No vacation |
|  |  |  |  | Not being able to enjoy summer |
|  |  |  |  | No real summer |
|  |  |  |  | No normal vacation |
|  |  |  |  | Summer plans cancelled |
|  |  |  |  | Not being able to enjoy weather with friends |
|  |  |  |  | Vacation not relaxing |
|  |  | **Closed public places** | 34 | No festivals, concerts, events |
|  |  |  |  | Gyms, pools closed, and sports trainings cancelled |
|  |  |  |  | Ban on meetings |
|  |  |  |  | No or only restricted visits allowed in hospitals |
|  |  |  |  | Library closed |
| Negative emotions | 93 | **Uncertainty in the moment (present) and regarding future** | 57 | Uncertainty (future, work, studies) |
|  |  |  |  | Uncertainty about how to act |
|  |  |  |  | Not being able to plan because of unpredictability |
|  |  |  |  | No end in sight |
|  |  |  |  | Moral obligations |
|  |  |  |  | Lack of control |
|  |  |  |  | Length of pandemic |
|  |  |  |  | The constantly changing guidelines of the health department |
|  |  |  |  | Constant changes in communicated facts |
|  |  | **Negative emotions** | 14 | Helplessness |
|  |  |  |  | Fear of virus |
|  |  |  |  | Depressive state |
|  |  |  |  | Listlessness |
|  |  |  |  | Guilt |
|  |  |  |  | Feeling of missing out |
|  |  | **Always having to be careful (tiring use of cognitive resources)** | 11 | Always having to be careful |
|  |  |  |  | Having to always think before doing something |
|  |  |  |  | Not knowing, what other people's attitudes are |
|  |  |  |  | Not being able to go anywhere carefree |
|  |  |  |  | Worry about not being careful enough |
|  |  |  |  | Other people's judgement when going out |
|  |  |  |  | Not being able to go on vacation carefree |
|  |  |  |  | Stressful to meet people |
|  |  |  |  | Having to be careful when going to the pool |
|  |  | **Worry about others** | 7 | Seeing how others are doing poorly |
|  |  |  |  | Worry about infecting relative |
|  |  | **Other people’s negative emotions which can be felt** | ≤5 | Negative attitude of people |
|  |  |  |  | Realizing that people become more aggressive and more stressed |
|  |  |  |  | Everybody's pessimism |
| Social distancing | 61 | **Social distancing** | 61 | Not seeing loved ones |
|  |  |  |  | Restricted social life |
|  |  |  |  | Not being able to hug loved ones |
|  |  |  |  | Loneliness/being alone |
|  |  |  |  | Ban on meetings |
|  |  |  |  | Missing human contact |
|  |  |  |  | Distance |
|  |  |  |  | Isolation |
|  |  |  |  | Not being close to friends |
|  |  |  |  | Not seeing fellow students |
|  |  |  |  | Couldn't do much with friends |
|  |  |  |  | Not being able to enjoy weather with friends |
| How politicians, media and others acted/handled the situation | 34 | **Disagreement, polarizations of opinions** | 16 | Divisions of opinions, disagreements |
|  |  |  |  | People's panic |
|  |  |  |  | More people believe in conspiracy theories |
|  |  |  |  | Seeing how stupid, naïve, and easy to manipulate people are |
|  |  |  |  | How the whole world is overreacting to a meaningless virus |
|  |  |  |  | People who don't take the pandemic seriously |
|  |  |  |  | Protests against measures |
|  |  |  |  | COVID deniers |
|  |  | **Poor handling of situation by media and politicians (criticism)** | 10 | Fearmongering of the media |
|  |  |  |  | Media |
|  |  |  |  | Censorship from the media |
|  |  |  |  | Exaggeration of media |
|  |  |  |  | The constantly changing guidelines of the health department |
|  |  |  |  | That clubs opened again |
|  |  |  |  | Mask obligation came too late |
|  |  |  |  | That people lost trust in the government |
|  |  | **How others acted/did or did not comply with measures** | 8 | People who don't stick to imposed measures |
|  |  |  |  | The behavior of others |
|  |  |  |  | Seeing the rise of authoritarianism |
| Changes to everyday life | 33 | **Changes to everyday life** | 17 | Missing structure to daily life and routine |
|  |  |  |  | COVID main topic of conversation |
|  |  |  |  | Constantly being at home |
|  |  |  |  | Not going outside |
|  |  |  |  | Overall COVID stress |
|  |  | **More bored during pandemic** | 11 | Boredom |
|  |  |  |  | Monotony |
|  |  |  |  | Lack of motivation |
|  |  |  |  | Doing nothing |
|  |  | **Less likely to be home alone during pandemic** | ≤5 | Everyone crammed together at home |
|  |  |  |  | COVID stress leads to conflict in the family |
|  |  |  |  | Less quiet and time for oneself at home due to family being at home |
| Changes to work/studies | 33 | **Changes to work/studies** | 33 | Exams postponed and having to learn in summer |
|  |  |  |  | Working from home |
|  |  |  |  | Quarantine during military service |
|  |  |  |  | Projects cancelled |
|  |  |  |  | Changes in studies |
|  |  |  |  | Exams |
|  |  |  |  | Cancelled semester abroad |
|  |  |  |  | Cancelled internship |
|  |  |  |  | Cancelled studies abroad |
| Damage to economy affecting livelihood | 28 | **Damage to one's own economic situation** | 14 | Financial difficulties |
|  |  |  |  | Lost work |
|  |  |  |  | Fear about losing work |
|  |  |  |  | Job change due to COVID |
|  |  | **Damage to the economic situation in general** | 14 | Unemployment |
|  |  |  |  | Seeing how others are losing their jobs |
|  |  |  |  | Effect on economy |
|  |  |  |  | Furlough/short-time work |
| Negative effect on relationships | 7 | **Negative effect on relationships** | 7 | Break-up |
|  |  |  |  | Difficult time for relationship |
|  |  |  |  | Difficult relationship between parents |
| Losing loved ones | 6 | **Losing friends** | ≤5 | Lost friends |
|  |  | **Death** | ≤5 | Death of relatives |
|  |  |  |  | Death of relative due to COVID |
| Other themes |  |  |  |  |
| Nothing | 9 |  |  | Nothing |

**Figure S1.** Word cloud with codes and frequency of coding, September 2020. Color-coded by overarching theme. Larger font size indicates greater frequency.

a) The best about the first “COVID-summer,” answers provided in mid-September 2020.


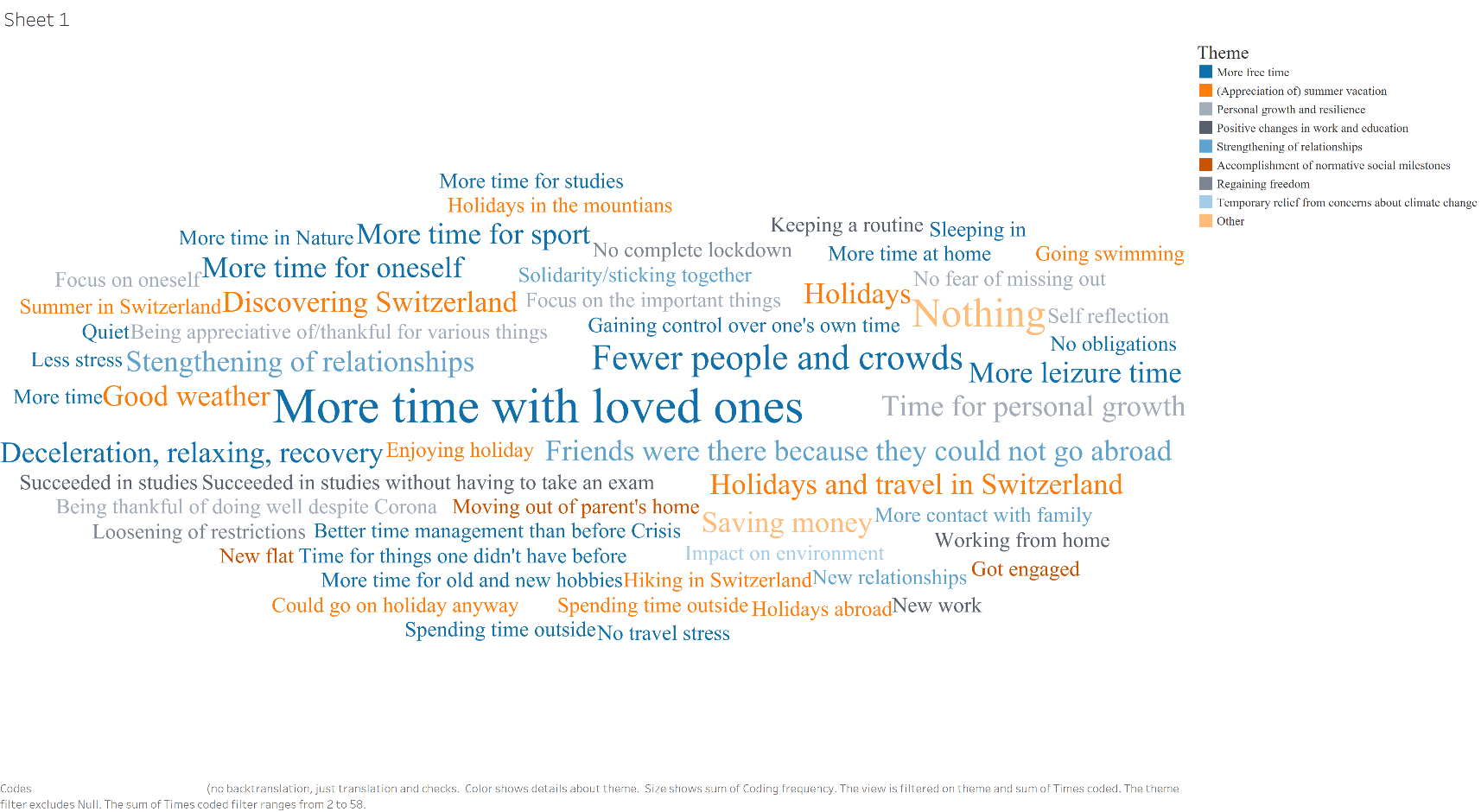

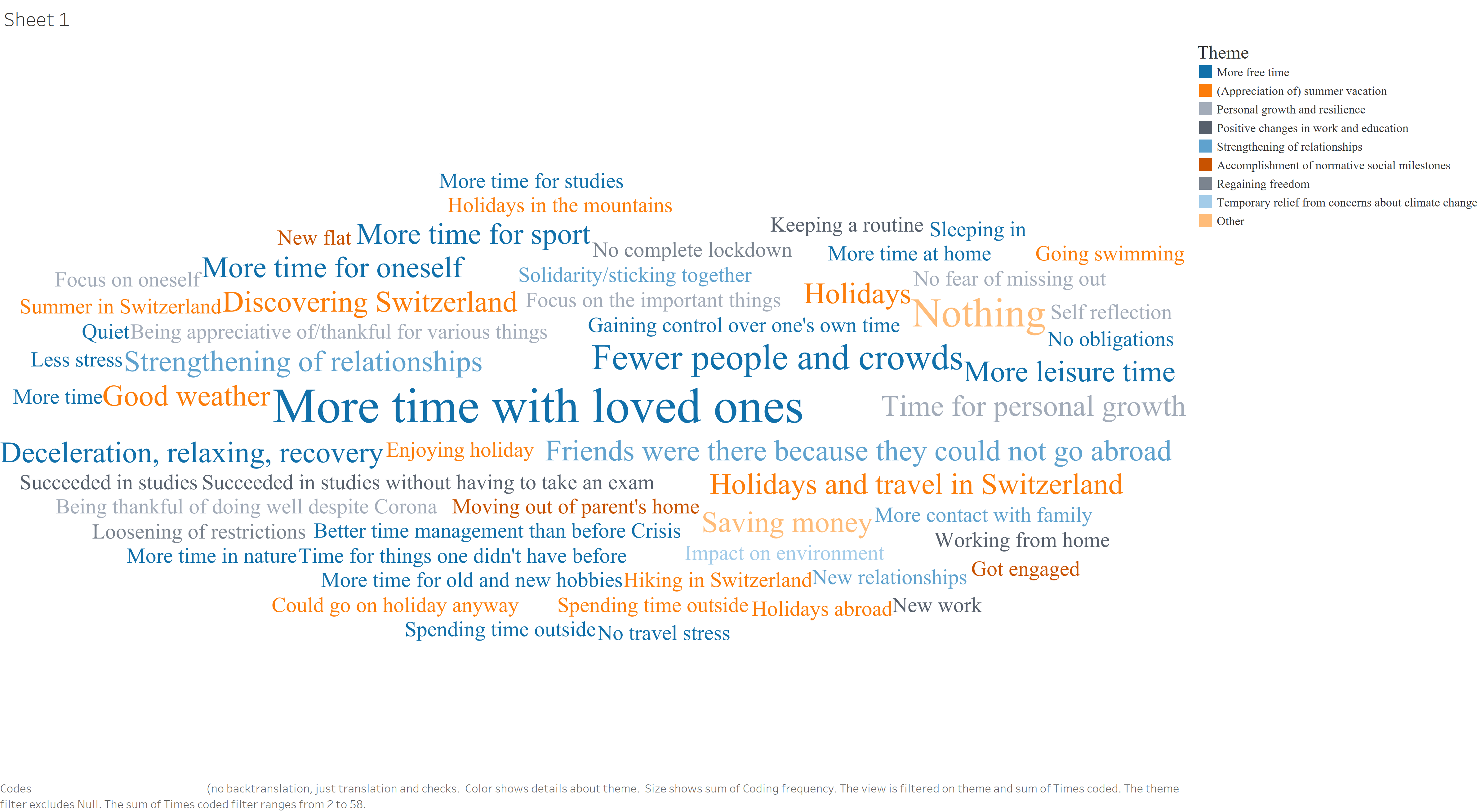


b) The worst about the first “COVID-summer,” answers provided in mid-September 2020.


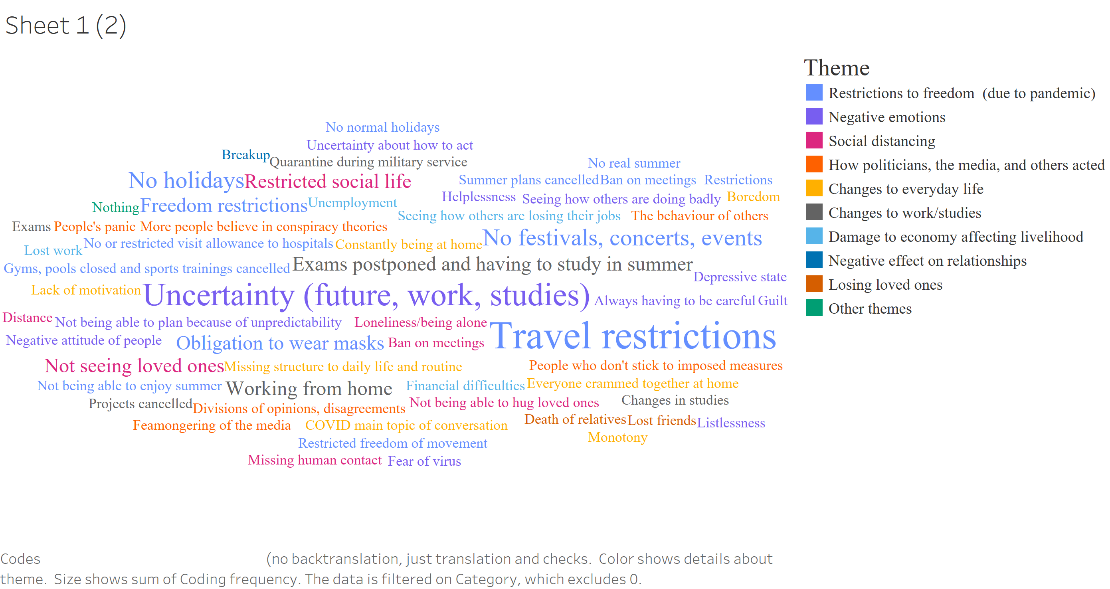

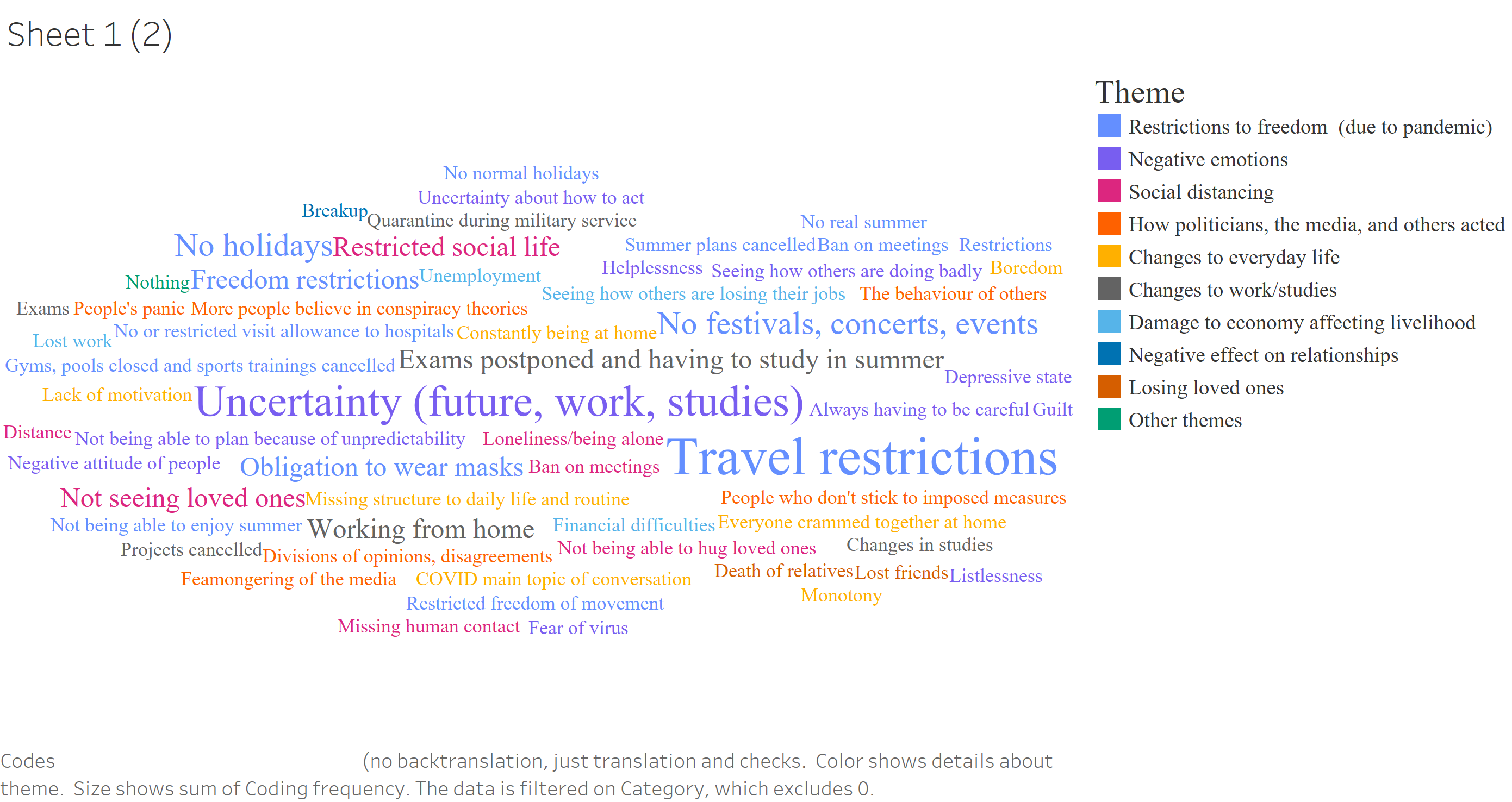


*Note:* Largest font size: Indicates 50+ responses. Medium font size: Indicates 20-29 responses. Smallest font size: Indicates 2-9 responses.
